# Transition Metal-Mediated Preparation of Nitrogen-Doped Porous Carbon for Advanced Zinc-Ion Hybrid Capacitors

**DOI:** 10.3390/nano15020083

**Published:** 2025-01-07

**Authors:** Mingcheng Li, Zheng Liu, Dan Wu, Huihao Wu, Kuikui Xiao

**Affiliations:** 1 Key Laboratory of Low Carbon and Environmental Functional Materials of College of Hunan Province, College of Materials and Chemical Engineering, Hunan City University, Yiyang 413000, China; limingcheng23@163.com (M.L.); wudanwd@hnu.edu.cn (D.W.); huihaowu1@gmail.com (H.W.); 2 Key Laboratory of Carbon Materials of Zhejiang Province, College of Chemistry and Materials Engineering, Wenzhou University, Wenzhou 325035, China

**Keywords:** zinc-ion hybrid capacitors, nitrogen-doped, metal-mediated, porous carbon

## Abstract

Carbon is predominantly used in zinc-ion hybrid capacitors (ZIHCs) as an electrode material. Nitrogen doping and strategic design can enhance its electrochemical properties. Melamine formaldehyde resin, serving as a hard carbon precursor, synthesizes nitrogen-doped porous carbon after annealing. Incorporating transition metal catalysts like Ni, Co, and Fe alters the morphology, pore structure, graphitization degree, and nitrogen doping types/proportions. Electrochemical tests reveal a superior capacitance of 159.5 F g^−1^ at a scan rate of 1 mV s^−1^ and rate performance in Fe-catalyzed N-doped porous carbon (Fe-NDPC). Advanced analysis shows Fe-NDPC’s high graphitic nitrogen content and graphitization degree, boosting its electric double-layer capacitance (EDLC) and pseudocapacitance. Its abundant micro- and mesopores increase the surface area fourfold compared to non-catalyzed samples, favoring EDLC and fast electrolyte transport. This study guides catalyst application in carbon materials for supercapacitors, illuminating how catalysts influence nitrogen-doped porous carbon structure and performance.

## 1. Introduction

With the large-scale integration of renewable energy, which is representative of new energy sources, into the power grid, energy storage technology has undergone significant development [1,2]. Despite these developments, energy storage technologies still face major limitations. Batteries boast high energy density but suffer from low power density and a limited cycle life [3,4,5]. Conversely, supercapacitors excel in high power density, a long cycle life, and rapid charging, yet they have low energy density, high self-discharge rates, and relatively high costs [6,7,8,9]. Zinc-ion hybrid capacitors (ZIHCs), however, combine the high energy density of batteries with the high power density of supercapacitors, enabling them to deliver substantial energy in a short period and to charge and discharge rapidly as needed while also exhibiting a long cycle life [10,11,12,13].

In ZIHCs, the cathode material significantly influences the performance [14,15,16,17]. Carbon materials are widely used in electrochemical energy storage systems due to their low cost, ease of preparation, high specific surface area, stable physicochemical properties, and good electrical conductivity [18,19,20,21]. Among carbon-based materials, porous carbon possesses a very high specific surface area and controllable pore structure, along with good stability, and so is regarded as a promising cathode material [22,23]. During charging and discharging, it provides channels and storage space for ion migration, but it has the limitation of low capacity [24]. Doping nitrogen atoms into the carbon matrix not only increases the number of active sites and electrical conductivity but also induces additional pseudocapacitance [25,26]. Additionally, it optimizes the morphology and pore structure of the carbon material to enhance wettability, thereby improving the electrochemical performance of the electrode material [27,28]. Therefore, nitrogen-doped porous carbon with a high surface area has recently been widely applied as the cathode in ZIHCs. For example, Zhang et al. [29] prepared nitrogen-doped lignin-derived porous carbon with a high specific surface area, leveraging the pseudocapacitance capacity brought about by high nitrogen doping. Qiu et al. [30] proposed a layered B/N co-doped porous carbon as the cathode for hybrid zinc-ion capacitors. Liu et al. [31] prepared nitrogen-doped porous carbon material with an ultra-high specific surface area (greater than 3000 m^2^ g^−1^) using sucrose as the carbon source, urea as the nitrogen source, and potassium oxalate as the activator, through simple grinding and high-temperature carbonization. Liu et al. [32] used chitosan as a carbon source, and ferric nitrate and potassium bicarbonate as porogens to prepare nitrogen-doped hierarchical porous carbon for zinc-ion hybrid capacitors. Although the above nitrogen-doped carbon materials have achieved certain performances, more efforts are needed on the effect of the intrinsic properties of the carbon on the ZIHCs. Firstly, these biomass carbon sources are directly converted into carbon at high temperature, and their morphology is difficult to change. Secondly, their nitrogen content and type are controlled by the main carbon source, which is difficult to regulate by effective means. In addition, these carbons lack an ordered carbon structure. Although some transition metals such as iron are added as catalysts to attempt to increase their degree of graphitization, these types of carbon sources cannot be reassembled from the atomic level, so it is difficult to generate highly graphitized carbon structures such as carbon nanotubes. More importantly, the mechanism by which microscopic factors such as nitrogen doping type and graphitization degree affect the performance of carbon materials in zinc-ion capacitors is still unclear.

Herein, an investigation has been conducted to explore the effects of the N-doped porous carbon (NDPC) with different catalysts during the thermal treatment process. Under the action of different catalysts, the physical and chemical properties of NDPC, such as specific surface area, nitrogen doping content and type, and degree of graphitization, show great differences. Results show that the NDPC catalyzed by Fe possesses the highest proportions in terms of the graphitic nitrogen, appropriate nitrogen content, and graphitization degree, as well as the largest surface area among all samples. With those qualities, the Fe-NDPC exhibits the highest capacity and fastest kinetics compared with the other samples.

## 2. Materials and Methods

### 2.1. Materials

Melamine, oxalic acid, ferric chloride, nickel chloride, cobalt chloride, tetraethyl orthosilicate, triethanolamine, formaldehyde, hydrofluoric acid, and hydrochloric acid were purchased from Aladdin Reagent Co., Ltd. (Shanghai, China). Octylphenol polyoxyethylene ether (OP-10), N-Methyl-2-pyrrolidone (NMP), and polyvinyl alcohol (PVA) were obtained from Sinopharm Chem. Reagent Co., Ltd. (Shanghai, China). Ketjen black, polyvinylidene fluoride (PVDF), and glass microfiber filters (GF/D, Whatman Co., Ltd., Maidstone, England) were purchased from Canrd New Energy Technology Co., Ltd. (Guangzhou, China). All chemicals were utilized without further modification.

### 2.2. Preparation of Fe-NDPC and Other Samples

Firstly, 0.075 mol melamine and 0.1 g PVA were placed in a beaker. To this was added 40 mL deionized water, 1.5 mL triethanolamine (10 wt %), and 15 mL formaldehyde. It was kept at 85 °C for 60 min to obtain Solution A. We dissolved 0.01 mol ferric chloride in 85 mL deionized water, and added 6 mL tetraethyl orthosilicate and 6 mL OP-10 (10 wt%). This was kept at 50 °C for 60 min to obtain Solution B. After cooling Solution A, it was poured into Solution B, 10 mL oxalic acid (1 M) was added, and it was kept at 50 °C for 2 h. This was then filtered, and dried at 100 °C for 12 h and 200 °C for 12 h to obtain the precursor. Heat treatment was carried out at 900 °C for two hours in a mixture of 20% hydrogen and 80% argon, with a heating rate of 5 °C per minute. Next, it was treated with hydrofluoric and hydrochloric acids to obtain Fe-NDPC. Using the same method, Ni-NDPC, Co-NDPC, and NDPC were prepared with nickel chloride, cobalt chloride, and a blank control, respectively.

### 2.3. Characterization

The morphologies and microstructures of the Fe-NDPC, Ni-NDPC, Co-NDPC, and NDPC were characterized by SEM (SEM5000, China instru & Quantum tech Co., Ltd., Hefei, China) and transmission electron microscopy (TEM, JEM2100F, JEOL, Tokyo, Japan), respectively. Raman spectroscopy was tested by a Raman spectrometer (NOVA2S, Shanghai Idea optics Corp., Ltd., Shanghai, China). X-ray diffraction (XRD) measurements were conducted using an XD6 X-ray powder diffractometer (Puxi General Instrument Co., Ltd., Beijing, China), employing Cu Kα radiation with a wavelength of λ = 0.154 nm. X-ray photoelectron spectroscopy (XPS) data were acquired using a Thermo Scientific K-Alpha (Thermo Fisher Scientific, Waltham, MA, USA). The Brunauer–Emmett–Teller (BET) surface area was determined using a Quantachrome Autosorb iQ (Quantachrome Instruments, Boynton Beach, FL, USA) via N_2_ adsorption/desorption experiments, and the nitrogen adsorption/desorption measurements were analyzed by the Barrett–Joyner–Halenda (BJH) method.

### 2.4. Electrochemical Measurements

Electrochemical performance of the obtained material was characterized by a two-electrode system with 2 M ZnSO_4_ as the electrolyte. Firstly, a slurry was prepared according to the mass ratio of carbon material–Ketjen black–PVDF as 8:1:1, with the solvent NMP. The resulting slurry was coated on a stainless steel mesh as the current collector using a four-sided coater, and after drying, the coated film was cut into discs (16 mm in diameter) using a punching machine to form the positive electrode pieces. Zn metal foil (0.1 mm thick; 16 mm in diameter) polished with fine sandpaper was directly used as the anode. Then, a zinc-ion hybrid capacitor was assembled in the order of a negative electrode shell, spring clip, gasket, negative electrode piece (zinc sheet), electrolyte, separator, electrolyte, positive electrode piece, and positive electrode shell, and sealed using a battery sealing machine for testing. Within a voltage range of 0.2~1.8 V, cyclic voltammetry curves at different scanning rates were tested; constant current charge–discharge curves at different current densities were tested within the same voltage window; and electrochemical impedance spectroscopy was conducted within a frequency range of 0.01 Hz~100 kHz. Detailed calculations of capacity are given in the Supporting Information.

## 3. Results and Discussion

**Figure 1:**
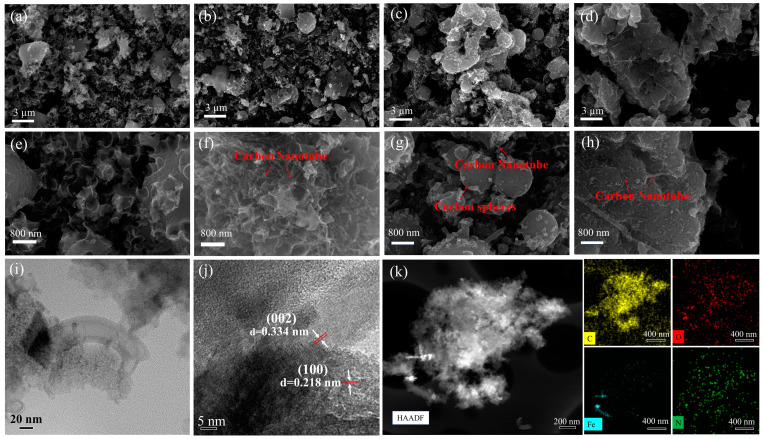
SEM images of (**a**,**e**) NDPC, (**b**,**f**) Ni-NDPC, (**c**,**g**) Co-NDPC, and (**d**,**h**) Fe-NDPC. (**i**) TEM image of Fe-NDPC. (**j**) TEM image of Fe-NDPC with lattice fringe. (**k**) HAADF-STEM image of Fe-NDPC and the corresponding EDS element mapping of C, O, Fe, and N, respectively.

Employing melamine as both the carbon and nitrogen sources, and incorporating different catalysts including Fe, Ni, and Co into the precursor, nitrogen-doped porous carbon materials were obtained after thermal treatment, which were denoted as Fe-NDPC, Co-NDPC, and Ni-NDPC, respectively. The SEM and EDS images of the as-prepared products are shown in Figure 1. The NDPC without a catalyst shows a wrinkled surface structure (Figure 1a,e). The Ni-NDPC possesses an overall blocky structure of uneven size, with carbon nanotubes randomly distributed (Figure 1b,f). Co-NDPC is composed of many carbon spheres and irregular platforms, with some small particles on the carbon spheres and a few carbon nanotubes interspersed among them (Figure 1c,g), and Fe-NDPC is composed of stacked carbon particles with carbon nanotubes on the surface (Figure 1d,h). From the above SEM results, it can be seen that adding different metals as catalysts during the preparation of carbon materials can yield carbon materials with distinctly different morphologies. The EDS spectra indicate that all four samples contain the elements C, N, O, and Si, and respective metal elements (Figure S1). Overall, the elemental distribution in the four samples is relatively uniform. In Figure 1j, the high-resolution transmission electron microscopy (HRTEM) of Fe-NDPC reveals lattice stripes in the crystalline regions, with the interlayer spacings d of the two regions calculated to be 0.334 nm and 0.218 nm, respectively, which are close to the lattice spacings of the (002) and (100) crystal planes of graphite, indicating partial graphitization of the carbon material. The structures of the materials can be more clearly distinguished from the TEM images (Figure S1e–g). The NDPC carbon is relatively smooth and no CNTs are found. Ni-NDPC has CNTs interspersed in the porous carbon like Fe-NDPC. For Co-NDPC, although a small amount of CNTs are generated, they are basically attached to the outside of the carbon structure and do not connect the materials well. The high-angle annular dark-field scanning transmission electron microscopy (HAADF-STEM) images and element mapping images in Figure 1k clearly show the distribution of C, O, N, and Fe elements and demonstrate the uniform distribution of N in Fe-NDPC.

Figure S2 presents the XRD patterns of the four samples, which shows that the NDPC exhibits a relatively broad diffraction peak at around 26°, corresponding to amorphous carbon. After catalysis with Fe, Co, and Ni, the graphite diffraction peak at 26° becomes significantly narrower, indicating that the catalysts have played a role in catalyzing graphitization, increasing the degree of graphitization of the carbon materials. Additionally, characteristic peaks of the elemental Fe, Co, and Ni are observed.

**Figure 2:**
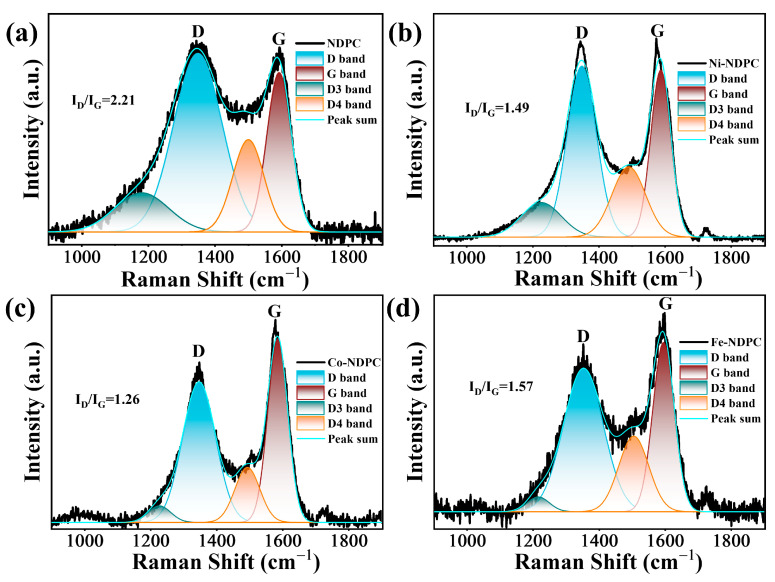
Raman spectrum of (**a**) NDPC, (**b**) Ni-NDPC, (**c**) Co-NDPC, and (**d**) Fe-NDPC.

Raman spectroscopy is used to analyze the degree of graphitization and defects in carbon materials (Figure 2). The D peak, located near 1350 cm^−1^, represents defects in the graphite lattice, disordered arrangements at edges, and low-symmetry carbon structures, also known as the structural disorder peak. The G peak, located near 1580 cm^−1^, corresponds to the inherent sp^2^ hybridized stretching vibration peak of natural graphite. By deconvolving the two broad signals in the Raman spectrum, two peaks at 1180 cm^−1^ (D3) and 1486 cm^−1^ (D4) can be obtained. The D3 peak is related to the stretching vibration of carbon atoms outside the graphene plane, while the D4 peak may be caused by doped nitrogen atoms [33]. The degree of graphitization of carbon materials can be quantitatively described using the ratio R (I_D_/I_G_) of the peak areas of the D peak and the G peak in the Raman spectrum [34]. A lower R value indicates a higher degree of graphitization, with fewer defects in the graphite-like lattice, disordered arrangements at edges, and low-symmetry carbon structures, and a more regular and ordered graphite-like lattice. After fitting and calculation, the R values for NDPC, Ni-NDPC, Co-NDPC, and Fe-NDPC are 2.21, 1.49, 1.26, and 1.57, respectively, indicating that the degree of graphitization of the materials increases after the addition of metal catalysis.

**Figure 3:**
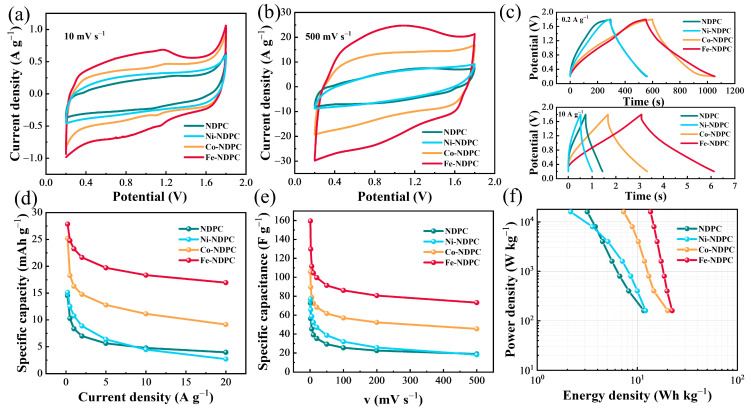
Electrochemical properties of the samples in 2 M ZnSO_4_. (**a**,**b**) CV patterns of NDPC, Ni-NDPC, Co-NDPC, and Fe-NDPC at scan rates of 10 and 500 mV s^−1^, respectively. (**c**) GCD curves at current densities of 0.2 A g^−1^ and 10 A g^−1^. (**d**) Rate performance at current densities from 0.1~20 A g^−1^. (**e**) Specific capacitance diagram at different scan rates. (**f**) Ragone plots.

To investigate the electrochemical performance of the samples, cyclic voltammetry (CV) was conducted on NDPC, Ni-NDPC, Co-NDPC, and Fe-NDPC at scan rates ranging from 1 to 500 mV s^−1^. At a scan rate of 10 mV s^−1^, Co-NDPC and Fe-NDPC exhibited more pronounced redox peaks than Ni-NDPC and NDPC (Figure 3a), suggesting obvious pseudocapacitive behavior and faster ion transport rates of Co-NDPC and Fe-NDPC at the low scan rate. Even at a higher scan rate of 500 mV s^−1^, the Fe-NDPC still exhibited clear redox peaks (Figure 3b), indicating it had better kinetics than the Co-NDPC. As the Fe-NDPC shows the largest areas in the CV curves, the capacity of the Fe-NDPC was significantly larger than the other three samples.

Galvanostatic charge–discharge (GCD) tests were also performed at current densities between 0.2 and 20 A g^−1^, where Fe-NDPC exhibited significantly better symmetry, closer to a standard inverted Y-shape, indicating a higher charge–discharge efficiency (Figure S3). Furthermore, Figure 3c compares the GCD curves of the four samples at current densities of 0.2 A g^−1^ and 10 A g^−1^. At the lower current density of 0.2 A g^−1^, Fe-NDPC and Co-NDPC exhibited similar capacities of 27.87 mAh g^−1^ and 25.22 mAh g^−1^, respectively. In contrast, NDPC and Ni-NDPC had significantly smaller capacities of 14.53 mAh g^−1^ and 15.12 mAh g^−1^, respectively. These performances are slightly inferior to Ni-Zn batteries [35] but better than asymmetric supercapacitors based on ZnCo_2_O_4_ [36]. When the current density increased to 10 A g^−1^, the capacity difference between Fe-NDPC and Co-NDPC widened, with Fe-NDPC having the highest capacity of 18.34 mAh g^−1^, followed by Co-NDPC at 11.13 mAh g^−1^, and NDPC and Ni-NDPC having even smaller capacities of 4.76 mAh g^−1^ and 4.45 mAh g^−1^, respectively. These results suggest that at low current densities, both Fe-NDPC and Co-NDPC exhibit higher capacities, while NDPC and Ni-NDPC have lower capacities, and this is in accordance with the CV results, which may be related to the pseudocapacitance generated by Fe- and Co-catalyzed samples. When further increasing the current density, the capacity of Fe-NDPC was much larger than that of Co-NDPC, consistent with the CV results at high scan rates. This may be related to its pore structure and N doping, which requires further characterization.

To more intuitively compare the performance differences among the samples, the specific capacities at different current densities (Figure 3d) and scan rates (Figure 3e) were calculated based on the GCD and CV curves, respectively. It can be concluded that the Fe-NDPC has a higher capacity and better performance, with a specific capacitance of up to 159.5 F g^−1^ at a scan rate of 1 mV s^−1^, and Co-NDPC and Ni-NDPC followed, while NDPC performed the worst, indicating that the addition of metal catalysis, especially Fe, can enhance the performance of the carbon electrode. The Ragone plot (Figure 3f) also shows that Fe-NDPC has high power density and energy density among the four samples. However, it cannot be ignored that when the current density is greater than 10 A g^−1^ or the scan rate is greater than 500 mV s^−1^, the specific capacity and specific capacitance of Ni-NDPC are smaller than those of NDPC. This phenomenon needs to be further analyzed through detailed structural characterization.

In general, carbon materials store charge through the synergistic effect of the co-adsorption mechanism and reversible adsorption/desorption of anions and cations at different potentials. The reversible diionic adsorption of cations and anions occurs in different potential ranges, and cation (Zn^2+^, H⁺) adsorption/desorption is the main process at low potentials, accompanied by the formation/dissolution of Zn_4_SO_4_(OH)_6_·5H_2_O (Equation (1)).

The reversible formation and dissolution of Zn_4_SO_4_(OH)_6_·5H_2_O are influenced by the adsorption of H^+^ ions. This adsorption and subsequent desorption process helps create a dynamic, locally alkaline environment close to the carbon electrode surface. In this alkaline setting, OH^−^ anions and Zn^2+^ ions form strong coordination bonds, leading to the synthesis of Zn_4_SO_4_(OH)_6_·5H_2_O. Furthermore, Zn_4_SO_4_(OH)_6_·5H_2_O, a significant Zn-containing compound, boosts the adsorption and storage capacity of Zn^2+^ ions on the carbon electrode. At high potentials, anion (SO_4_^2−^) adsorption/desorption is the main process [16,37].

4Zn^2+^ + 6OH^−^ + SO_4_^2−^ + 5H_2_O ↔ Zn_4_SO_4_(OH)_6_·5H_2_O↓ (1)

**Figure 4:**
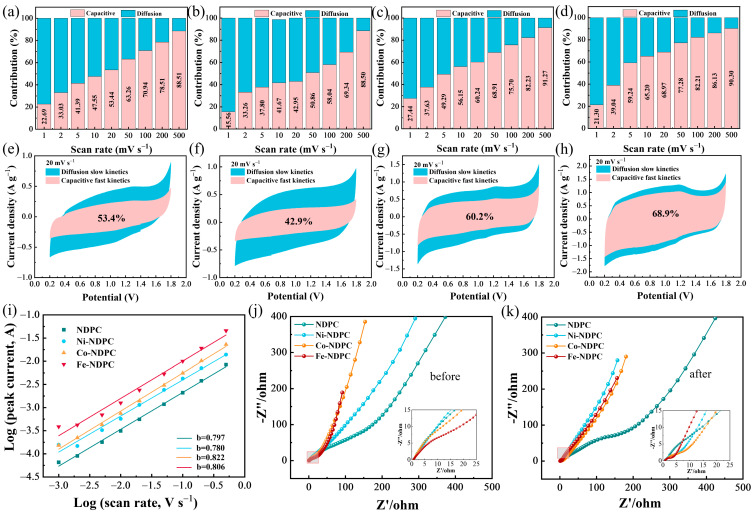
The contribution of the capacitive and diffusion control at different scan rates and the contribution of the surface process at CV scan rate of 20 mV s^−1^ for (**a**,**e**) NDPC, (**b**,**f)** Ni-NDPC, (**c**,**g**) Co-NDPC, and (**d**,**h**) Fe-NDPC, respectively. (**i**) Fitting curves of b values; Nyquist curves before (**j**) and after (**k**) cycle testing.

The CV and Nyquist curves were further utilized to evaluate the diffusion kinetics of the samples. The kinetics of electrolyte ions in ZIHCs were estimated using the formula: *i* = *av^b^*, where *i* is the peak current and *v* is the scan rate, and *a* and *b* are both relative constants. The contribution of the capacity arising from the diffusion process to the total capacity can be calculated by the Equations (S5)–(S7) [38,39]. Figure 4a–d present the capacitive and diffusion-controlled plots of the four samples at different scan rates. As the scan rate increases, the proportion of diffusion-controlled capacity gradually decreases, while the proportion of capacitively controlled capacity increases, which is due to the latter contributing more capacity due to their faster kinetics at higher scan rates [40]. Specifically, Fe-NDPC has the highest proportions of capacitively controlled capacity, which are 21.30%, 39.04%, 59.24%, 65.20%, 68.97%, 77.28%, 82.21%, 86.13%, and 90.30% at scan rates of 1, 2, 5, 10, 20, 50, 100, 200, and 500 mV s^−1^, respectively. Following Fe-NDPC are Co-NDPC, NDPC, and Ni-NDPC in descending order. Figure 4e–h show the CV curves and comparisons of capacitive and pseudocapacitive contributions for the four samples at a scan rate of 20 mV s^−1^. The higher proportion in the capacitively controlled capacity is beneficial for the fast energy storage, which can explain the better performance achieved at the higher scan rate. Figure 4i presents a regression plot of the b value, derived from the relationship between the peak current and scan rate. In general, the b value approximating 0.5 suggests a diffusion-controlled electrochemical process, whereas the b value nearing 1 signifies a predominantly surface-controlled capacitive behavior. It can be seen from the figure that the b value of Fe-NDPC is slightly smaller than that of Co-NDPC. This may be because Fe-NDPC produces a larger pseudocapacitance at a low scan rate, causing the peak current value at a low scan rate to deviate from the straight line. To further study the electrochemical performance of the samples, the cycle stability and coulombic efficiency were tested at 5 A g^−1^ (Figure S4). It can be found that after 3000 cycles, except for the decrease in capacity of Ni-NDPC, the capacity retention rate and coulombic efficiency of the other three are close to 100%, which may be related to the high metal and oxygen content in Ni-NDPC (Table S1). During the cycle, more redox reactions may occur, causing the zinc negative electrode to produce dendrites and lead to capacity decay. The metal content in Fe-NDPC is relatively small, so it can maintain a high cycle stability. In order to clarify the connection, a chemical impedance spectroscopy test was carried out. In the Nyquist plot (Figure 4j,k), the x-axis intercept in the high-frequency region represents the internal resistance (R_s_) of the electrode material, while the semicircle in the mid-frequency region indicates the charge transfer resistance (R_ct_). The slope of the impedance spectrum in the low-frequency region can reflect the surface process of the electrode. The closer it is to the x-axis, the closer it is to the ideal double-layer capacitor behavior [41]. The low-frequency slope of Ni-NDPC increases significantly after cycling, which indicates that the double-layer capacitance behavior of Ni-NDPC is slowly increasing during the cycling process. The low-frequency slope of Ni-NDPC increases significantly after cycling, which indicates that the double-layer capacitance of Ni-NDPC increases slowly during cycling. This also verifies the previous speculation that some metal or oxygen-containing groups in Ni-NDPC gradually fail during cycling, causing Ni-NDPC to eventually show obvious double-layer capacitance behavior. Before the cycling test, the R_s_ of NDPC, Ni-NDPC, Co-NDPC, and Fe-NDPC were 1.86 Ω, 0.74 Ω, 0.59 Ω, and 0.69 Ω, respectively, suggesting that the addition of metal catalysis significantly affects the internal resistance of the material. After the cycling test, the R_s_ of NDPC, Ni-NDPC, Co-NDPC, and Fe-NDPC slowly increased to 2.34 Ω, 0.79 Ω, 1.26 Ω, and 0.76 Ω. It is noteworthy that the Rs of Ni-NDPC and Fe-NDPC increased less compared with those of NDPC and Co-NDPC, which indicates that CNTs play a key role. The distribution of CNTs in Ni-NDPC and Fe-NDPC is relatively uniform and runs through the entire carbon substrate, which makes the internal resistance of the material stable during the cycle test. In the mid-frequency region, NDPC exhibits a relatively prominent semicircle, while Ni-NDPC, Co-NDPC, and Fe-NDPC show no obvious semicircles before the cycle test, indicating that the incorporation of metal elements can reduce the impedance of charge transfer resistance. From the enlarged image, it can be found that before cycling, Fe-NDPC seems to show a higher R_ct_ than the other samples, which may be related to the insufficient infiltration of the electrolyte at the beginning. After cycling, Fe-NDPC shows a smaller R_ct_ than the other samples. Bode plots are used to further understand the ideal double-layer and pseudocapacitive behaviors of materials (Figure S5). In the Bode plots, the closer the phase angle is to −90° at low frequencies, the closer it is to the ideal double-layer capacitance behavior. The phase angles of Ni-NDPC, Co-NDPC, and Fe-NDPC are all around −60° at low frequencies, indicating that they are all dominated by mixed capacitance. The phase angle of NDPC deviates further from −90° than the other three samples, indicating that it has the highest proportion of pseudocapacitance among the four samples, which is consistent with the results of the previous CV calculation. At a phase angle of −45°, the characteristic frequency f_0_ signifies the equality of resistive and capacitive impedances, and the corresponding time constant τ_0_ (which is defined as 1/f_0_) marks the transition point between the supercapacitor’s resistive behavior at frequencies above f_0_ and its capacitive behavior at frequencies below f_0_. Fe-NDPC has the smallest τ_0_ among them, further proving that Fe-NDPC has the highest electronic transport properties.

The porosity characteristics and specific surface areas of the NDPC, Ni-NDPC, Co-NDPC, and Fe-NDPC samples were characterized using N_2_ adsorption/desorption isotherms (Figure S6). It can be observed that the pore sizes of the four materials are mainly concentrated around 3.5 nm (inset of Figure S6). Previous studies have shown that optimal performance of supercapacitors can be achieved when the average micropore size in the nanostructured carbon matches the size of the ions in the electrolyte [42]. Compared to NDPC, the pore structures in Ni-NDPC, Co-NDPC, and Fe-NDPC show sharp peaks appearing at 1 nm, which can increase the surface area, offering improved capacitively controlled capacity. Additionally, Fe-NDPC exhibits a large number of mesopores around 8.5 nm, providing numerous channels for subsequent ion transport during charging and discharging. The specific surface area of NDPC is only 109.35 m^2^ g^−1^, while the specific surface areas of the samples with Ni, Co, and Fe, increase to 266.89, 371.67, and 531.03 m^2^ g^−1^, respectively. The Fe-NDPC not only possesses rich micro- and mesopores, but its specific surface area is also more than fourfold that of the NDPC, and these are key intrinsic reasons for the significant improvement in material performance.

**Figure 5:**
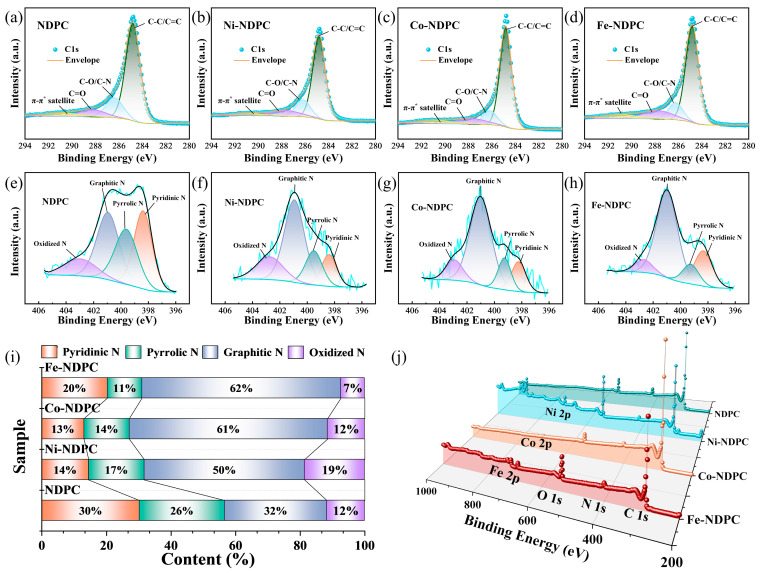
High-resolution C 1s and N 1s XPS spectra of (**a**,**e**) NDPC, (**b**,**f**) Ni-NDPC, (**c**,**g**) Co-NDPC, (**d**,**h**) Fe-NDPC, respectively. (**i**) Comparison of the different nitrogen contents of the samples and (**j**) XPS survey spectra.

To explore the surface elemental valence and electronic states of the materials, further investigation was conducted on different samples through XPS measurements. As shown in Figure 5a–d, the C 1s spectrum of NDPC, Ni-NDPC, Co-NDPC, and Fe-NDPC are divided into four parts, related to the C-C/C=C (284.8 eV), C-N/C-O (286.3 eV), C=O (288.2 eV), and π-π* conjugation bonds [43], respectively. The proportion of each C bond content is shown in Table S2. It can be found that the C=O bond content in Fe-NDPC is the highest. Oxygen atoms, characterized by high electronegativity and partial negative charge in C=O functional groups, exhibit a propensity for Zn^2+^ adsorption due to the capacity of Zn’s empty 3d orbital to engage in d-π conjugation with the π bond of C=O. This interaction leads to the generation of extra-domain electrons, thereby inducing pseudocapacitance and enhancing the specific capacitance of the electrode material [23,44]. High-resolution N 1s spectra were used to characterize the bonding configurations of nitrogen atoms (Figure 5e–h). Specifically, pyridinic nitrogen is located at 398.3~398.6 eV, pyrrolic nitrogen at 399.2~399.8 eV, graphitic nitrogen at 400.8~401.2 eV, and oxidized nitrogen at around 402.9~403.3 eV, respectively [45].

**Scheme 1:**
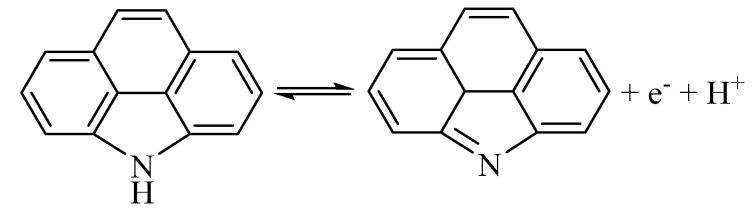
Faradaic redox reactions.

As shown in Figure 5i, the proportion of graphitic nitrogen in the Fe-NDPC is 62%, which is the highest among the four samples, followed by Co-NDPC (61%) then Ni-NDPC (50%) and NDPC (32%). Interestingly, the proportion of pyrrolic nitrogen is exactly the opposite, with the NDPC having the highest pyrrolic nitrogen content. Although studies have shown that pyridinic nitrogen and pyrrolic nitrogen can increase the electron density of states of the material more than graphitic nitrogen, thereby reducing the impact of quantum capacitance on capacitance [42,43], it seems that graphitic nitrogen can significantly enhance the charge transfer rate at the electrode/electrolyte interface, thereby optimizing capacitive performance, especially the contribution of the surface process. A DFT calculation shows that the pyrrolic nitrogen in the carbon skeleton has moderate adsorption energy and the most adsorption sites for zinc ions, thus enabling carbon with the same surface area to have more charge storage sites [23]. In other words, graphitic nitrogen favors double-layer energy storage, while pyridinic and pyrrolic nitrogen tends to make carbon materials exhibit pseudocapacitive energy storage. The zinc sulfate electrolyte is weakly acidic, and pyrrolic nitrogen can undergo a reversible storage of protons, forming the reversible Faradaic redox reactions (Scheme 1), thereby generating pseudocapacitance [46,47]. This mechanism matches the zinc ion storage mechanism mentioned above (Equation (1)) [23].

Figure 5j is the full XPS spectrum of the sample. It can be seen from the figure that there are obvious Fe 2p and Ni 2p peaks in Fe-NDPC and Ni-NDPC, respectively, while only a weak Co 2p peak can be seen in Co-NDPC. This may be related to the distribution of metals in the material. Co is more exposed on the surface of the carbon material and is cleaned during the acid treatment process, while Ni and Fe are more embedded in the interior of the carbon material. In particular, the catalyst particles that catalyze the growth of carbon nanotubes are tightly wrapped by the carbon layer and retained. Table S1 gives the total content of each element in the sample. It is not difficult to find that NDPC has the highest nitrogen content. With the addition of metal elements, the nitrogen content decreases, while Fe-NDPC has a relatively higher total nitrogen content than Ni-NDPC and Co-NDPC. Since Fe-NDPC has the appropriate nitrogen content, and the highest proportions of graphitic nitrogen and C=O bonds, it explains its higher capacity and rate capabilities in the above electrochemical tests. In order to better measure the influence of nitrogen doping content and specific surface area on capacitance, we normalized the specific capacitance to the specific surface area to obtain the area-normalized capacitance (Table S3). As can be seen from Table S3, whether at low or high scan rates or low or high current densities, the area-normalized capacitance of NDPC is always the largest, which indicates that nitrogen doping content can effectively increase the capacitance per unit surface area. Minakshi et al. [48] showed through DFT calculations that the introduction of N doping can promote the adsorption of O atoms on the graphite surface due to the charge transfer mechanism. In addition, electronic structure analysis showed that the orbital hybridization between the adsorbate and the graphite surface is enhanced when N doping is present. This may be one of the main reasons why nitrogen doping can improve area-specific capacitance. In summary, the specific surface area and pore structure of carbon materials play a key role in charge storage and ion transport. Under the same conditions, the nitrogen doping content and doping type will further optimize the charge storage per unit area, thereby further improving the charge storage capacity of nitrogen-doped carbon materials.

**Figure 6:**
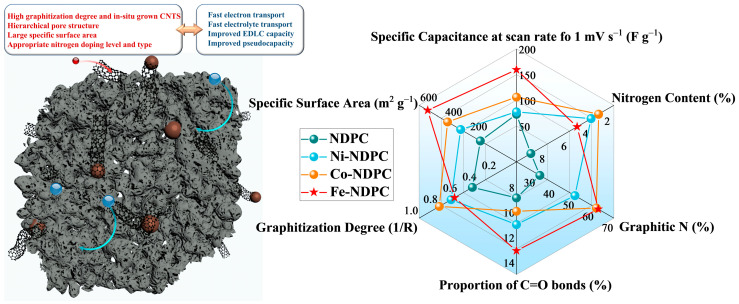
(**a**) Surface diagram of Fe-NDPC. (**b**) Radar figure.

Overall, as shown in Figure 6, transition metals can effectively catalyze the graphitization of polymer-derived carbon, but the content of doped nitrogen atoms decreases with increasing degree of graphitization. Fe-NDPC possesses an appropriate graphitization degree, nitrogen content with twining CNT, abundant micro–mesopore structure, the largest specific surface area, and the highest proportions of graphitic nitrogen and C=O bonds, resulting in fast electron and electrolyte transport, and finally exhibits improved EDLC and pseudocapacitance performance.

## 4. Conclusions

This work explores the impact of different catalysts, including Ni, Co, and Fe, on the catalytic graphitization of nitrogen-doped carbon materials and their performance in zinc-ion supercapacitors. Among these three transition metals, Co appears to be more conducive to the formation of graphitized carbon, Ni is beneficial for catalyzing the growth of carbon nanotubes, and Fe can produce more porous structures and a larger specific surface area while catalyzing the growth of carbon nanotubes. The good zinc ion capacitor performance of carbon materials is not solely determined by a single factor. Factors such as graphitization degree, specific surface area, and nitrogen doping content and type may be contradictory in some cases. Only by comprehensively balancing the advantages of all aspects can the best performance be obtained. The results indicate that the material obtained with the Fe catalyst exhibits high degree of graphitization and the highest content of graphitic nitrogen and C=O bonds, as well as the largest specific surface area. Due to these comprehensive physicochemical properties, the carbon material catalyzed by Fe exhibits the highest specific capacitance and best rate capability in electrochemical performance tests. This work provides a reference for the development of catalysts in the catalytic graphitization of carbon materials.

## Data Availability

Data are contained within this article or the Supplementary Materials.

## Supplementary Materials

The following supporting information can be downloaded at: https://www.mdpi.com/article/10.3390/nano15020083/s1, Figure S1: EDS-mapping images of (a) NDPC, (b) Ni-NDPC, (c) Co-NDPC, and (d) Fe-NDPC; TEM image of (e) NDPC, (f) Ni-NDPC, (g) Co-NDPC; Figure S2: XRD spectra of the four samples; Figure S3: Cyclic voltammograms of (a) NDPC, (b) Ni-NDPC, (c) Co-NDPC, and (d) Fe-NDPC at 1–500 mV s^−1^. CP curves at different current densities from 0.2 to 20 A g^−1^ for (e) NDPC, (f) Ni-NDPC, (g) Co-NDPC, and (h) Fe-NDPC; Figure S4: Cycle stability at 5 A g^−1^ of the samples. Figure S5: Bode plots of phase angle versus frequency. Figure S6: N_2_ adsorption/desorption isotherm of (a) NDPC, (b) Ni-NDPC, (c) Co-NDPC, and (d) Fe-NDPC; Table S1: The content of various elements in the samples; Table S2: The proportion of carbon bonds in the samples. Table S3: The area normalized capacitance of the samples. (See Refs. [39,49]).

## References

[1] Yang Z., Lv C., Li W., Wu T., Zhang Q., Tang Y., Shao M., Wang H. (2021). Revealing the Two-Dimensional Surface Diffusion Mechanism for Zinc Dendrite Formation on Zinc Anode. Small.

[2] Ma Y., Ge Y., Gao D., Li Z., Du M., Xing X., Feng R., Han Y. (2023). The structural transformation of metal-organic frameworks towards 2D carbon for a desirable supercapacitor. J. Mater. Chem. C.

[3] Blanc L.E., Kundu D., Nazar L.F. (2020). Scientific Challenges for the Implementation of Zn-Ion Batteries. Joule.

[4] Tang H., Yao J., Zhu Y. (2021). Recent Developments and Future Prospects for Zinc-Ion Hybrid Capacitors: A Review. Adv. Energy Mater..

[5] Mageto T., Bhoyate S.D., Mensah-Darkwa K., Kumar A., Gupta R.K. (2023). Development of high-performance zinc-ion batteries: Issues, mitigation strategies, and perspectives. J. Energy Storage.

[6] Shao Y., El-Kady M.F., Sun J., Li Y., Zhang Q., Zhu M., Wang H., Dunn B., Kaner R.B. (2018). Design and Mechanisms of Asymmetric Supercapacitors. Chem. Rev..

[7] Chakraborty S., Mary N.L. (2022). Review—An Overview on Supercapacitors and Its Applications. J. Electrochem. Soc..

[8] Zhang L., Wu D., Wang G., Xu Y., Li H., Yan X. (2021). An aqueous zinc-ion hybrid super-capacitor for achieving ultrahigh-volumetric energy density. Chin. Chem. Lett..

[9] Qiu D., Yue C., Qiu C., Xian L., Li M., Wang F., Yang R. (2022). Three-dimensional nitrogen-doped dual carbon network anode enabling high-performance sodium-ion hybrid capacitors. Electrochim. Acta.

[10] Huang Z., Zhang R., Zhang S., Li P., Li C., Zhi C. (2022). Recent advances and future perspectives for aqueous zinc-ion capacitors. Mater. Futures.

[11] Li H., Ma L., Han C., Wang Z., Liu Z., Tang Z., Zhi C. (2019). Advanced rechargeable zinc-based batteries: Recent progress and future perspectives. Nano Energy.

[12] Yin J., Zhang W., Alhebshi N.A., Salah N., Alshareef H.N. (2021). Electrochemical Zinc Ion Capacitors: Fundamentals, Materials, and Systems. Adv. Energy Mater..

[13] Catarineu N.R., Lin D., Zhu C., Oyarzun D.I., Li Y. (2023). High-performance aqueous zinc-ion hybrid capacitors based on 3D printed metal-organic framework cathodes. Chem. Eng. J..

[14] Huang Z., Wang T., Song H., Li X., Liang G., Wang D., Yang Q., Chen Z., Ma L., Liu Z. (2020). Effects of Anion Carriers on Capacitance and Self-Discharge Behaviors of Zinc Ion Capacitors. Angew. Chem. Int. Ed..

[15] Liu Y., Wu L. (2023). Recent advances of cathode materials for zinc-ion hybrid capacitors. Nano Energy.

[16] Wang Y., Sun S., Wu X., Liang H., Zhang W. (2023). Status and Opportunities of Zinc Ion Hybrid Capacitors: Focus on Carbon Materials, Current Collectors, and Separators. Nano-Micro Lett..

[17] Sun Z., Chu S., Jiao X., Li Z., Jiang L. (2024). Research progress of carbon cathode materials for zinc-ion capacitors. J. Energy Storage.

[18] Xue B., Xu J., Xiao R. (2023). Ice template-assisting activation strategy to prepare biomass-derived porous carbon cages for high-performance Zn-ion hybrid supercapacitors. Chem. Eng. J..

[19] Zhang D., Li L., Gao Y., Wu Y., Deng J. (2021). Carbon-Based Materials for a New Type of Zinc-Ion Capacitor. ChemElectroChem.

[20] Minakshi M., Samayamanthry A., Whale J., Aughterson R., Shinde P.A., Ariga K., Kumar Shrestha L. (2024). Phosphorous–Containing Activated Carbon Derived From Natural Honeydew Peel Powers Aqueous Supercapacitors. Chem.—Asian J..

[21] Minakshi M., Mujeeb A., Whale J., Evans R., Aughterson R., Shinde P.A., Ariga K., Shrestha L.K. (2024). Synthesis of Porous Carbon Honeycomb Structures Derived from Hemp for Hybrid Supercapacitors with Improved Electrochemistry. ChemPlusChem.

[22] Zhang W., Cheng R.-R., Bi H.-H., Lu Y.-H., Ma L.-B., He X.-J. (2021). A review of porous carbons produced by template methods for supercapacitor applications. New Carbon Mater..

[23] Xu Z., Sun Z., Shan J., Jin S., Cui J., Deng Z., Seo M.H., Wang X. (2023). O, N-Codoped, Self-Activated, Holey Carbon Sheets for Low-Cost And High-Loading Zinc-Ion Supercapacitors. Adv. Funct. Mater..

[24] Du W., Ang E.H., Yang Y., Zhang Y., Ye M., Li C.C. (2020). Challenges in the material and structural design of zinc anode towards high-performance aqueous zinc-ion batteries. Energy Environ. Sci..

[25] Li J., Zhang J., Yu L., Gao J., He X., Liu H., Guo Y., Zhang G. (2021). Dual-doped carbon hollow nanospheres achieve boosted pseudocapacitive energy storage for aqueous zinc ion hybrid capacitors. Energy Storage Mater..

[26] Liu L., Sun X., Dong Y., Wang D., Wang Z., Jiang Z., Li A., Chen X., Song H. (2021). N-doped hierarchical porous hollow carbon spheres with multi-cavities for high performance Na-ion storage. J. Power Sources.

[27] Yin J., Zhang W., Wang W., Alhebshi N.A., Salah N., Alshareef H.N. (2020). Electrochemical Zinc Ion Capacitors Enhanced by Redox Reactions of Porous Carbon Cathodes. Adv. Energy Mater..

[28] Liu Q., Zhang H., Xie J., Liu X., Lu X. (2020). Recent progress and challenges of carbon materials for Zn-ion hybrid supercapacitors. Carbon Energy.

[29] Zhang W., Yin J., Jian W., Wu Y., Chen L., Sun M., Schwingenschlögl U., Qiu X., Alshareef H.N. (2022). Supermolecule-mediated defect engineering of porous carbons for zinc-ion hybrid capacitors. Nano Energy.

[30] Lu Y., Li Z., Bai Z., Mi H., Ji C., Pang H., Yu C., Qiu J. (2019). High energy-power Zn-ion hybrid supercapacitors enabled by layered B/N co-doped carbon cathode. Nano Energy.

[31] Liu Z.M., Zhong W.S., Li J.X., Hu G.S. (2024). Preparation of nitrogen-doped porous carbons with ultra-highsurface areas for high-performance supercapacitors. Chin. J. Inorg. Chem..

[32] Liu P., Gao Y., Tan Y., Liu W., Huang Y., Yan J., Liu K. (2019). Rational design of nitrogen doped hierarchical porous carbon for optimized zinc-ion hybrid supercapacitors. Nano Res..

[33] Liu Z., Xiao K., Guo H., Ning X., Hu A., Tang Q., Fan B., Zhu Y., Chen X. (2017). Nitrogen-Doped Worm-Like Graphitized Hierarchical Porous Carbon Designed for Enhancing Area-Normalized Capacitance of Electrical Double Layer Supercapacitors. Carbon.

[34] Chen X., Zhang Y., Tao L., Nie Q., Meng F., Zhu S., Cui L., Huang R. (2020). Ferromagnetic carbonized polyaniline/nanodiamond hybrids for ultrabroad-band electromagnetic absorption. Carbon.

[35] Ayyanusamy P., Swathi T.D., Alphonse R., Minakshi M., Sivasubramanian R. (2024). Synthesis of Amorphous Nickel-Cobalt Hydroxides for Ni−Zn Batteries. Chem.—A Eur. J..

[36] Haripriya M., Manimekala T., Dharmalingam G., Minakshi M., Sivasubramanian R. (2024). Asymmetric Supercapacitors Based on ZnCo_2_O_4_ Nanohexagons and Orange Peel Derived Activated Carbon Electrodes. Chem.—Asian J..

[37] Wang L., Peng M., Chen J., Hu T., Yuan K., Chen Y. (2022). Eliminating the Micropore Confinement Effect of Carbonaceous Electrodes for Promoting Zn-Ion Storage Capability. Adv. Mater..

[38] Hu W., Chen L., Geng B., Du M., Shan G., Song Y., Wu Z., Zheng Q. (2023). Coral-Inspired Hierarchical Structure Promotes a Win-Win on Mass Loading and Rate Capability: A Nickel-Cobalt-Zinc-Sulfide@Nickel-Cobalt Layered Double Hydroxide Electrode for a High-Performance Hybrid Supercapacitor. ACS Appl. Energy Mater..

[39] Gao T., Luo W., Yang Y., Zhou Y., Xu J., Li N., Li J., Liu Z. (2024). Engineering hierarchically porous carbon nanorods electrode materials for high performance zinc ion hybrid supercapacitors. Colloids Surf. A.

[40] Dong L., Yang W., Yang W., Wang C., Li Y., Xu C., Wan S., He F., Kang F., Wang G. (2019). High-Power and Ultralong-Life Aqueous Zinc-Ion Hybrid Capacitors Based on Pseudocapacitive Charge Storage. Nano-Micro Lett..

[41] Yang B., Zhao W., Gao Z., Yang J., Shi W., Zhang Y., Su Q., Xu B., Du G. (2024). Flexible CNT@Porous carbon sponge cathode with large mesopores for high-rate zinc-ion hybrid capacitors. Carbon.

[42] Zhu J., Childress A.S., Karakaya M., Dandeliya S., Srivastava A., Lin Y., Rao A.M., Podila R. (2016). Defect-Engineered Graphene for High-Energy- and High-Power-Density Supercapacitor Devices. Adv. Mater..

[43] Zhang Z., Deng S., Wang D., Qing Y., Yan G., Li L., Wu Y. (2023). Low-tortuosity carbon electrode derived from Wood@ZIF-67 for supercapacitor applications. Chem. Eng. J..

[44] Jiang J., Yao L., Peng H., Wei G., Tian Y., Sun L., Dai P., Cai P., Zou Y., Zhang H. (2024). High-Performance Zinc-Ion Hybrid Supercapacitor from Guilin Sanhua Liquor Lees-Derived Carbon Materials. ACS Appl. Mater. Interfaces.

[45] Zhao F.-J., Zhu Y., Chen Y., Ren X.-Y., Dong H., Zhang H., Ren Q., Luo H.-B., Zou Y., Ren X.-M. (2024). Acidified Nitrogen Self-Doped Porous Carbon with Superprotonic Conduction for Applications in Solid-State Proton Battery. Small.

[46] Tian K., Wang J., Cao L., Yang W., Guo W., Liu S., Li W., Wang F., Li X., Xu Z. (2020). Single-site pyrrolic-nitrogen-doped sp2-hybridized carbon materials and their pseudocapacitance. Nat. Commun..

[47] Wei J.-S., Ding H., Wang Y.-G., Xiong H.-M. (2015). Hierarchical Porous Carbon Materials with High Capacitance Derived from Schiff-Base Networks. ACS Appl. Mater. Interfaces.

[48] Wickramaarachchi K., Minakshi M., Aravindh S.A., Dabare R., Gao X., Jiang Z.-T., Wong K.W. (2022). Repurposing N-Doped Grape Marc for the Fabrication of Supercapacitors with Theoretical and Machine Learning Models. Nanomaterials.

[49] Gao P., Shen B., Zhao P., Shi G., Zhao X. (2023). Tuning the Mn^2+^/Mn^3+^ ratio of ZnMn_2_O_4_ from spent zinc-carbon battery powder to enhance the electrochemical performance. J. Power Sources.

