# Supplementary material for: Transition Metal-Mediated Preparation of Nitrogen-Doped Porous Carbon for Advanced Zinc-Ion Hybrid Capacitors
Source: Nanomaterials (Basel). 2025 Jan 7;15(2):83. doi: 10.3390/nano15020083 (PMC11767337; doi:10.3390/nano15020083)
Supplement: Supplementary file 1 [file nanomaterials-15-00083-s001.zip › nanomaterials-3406376-supplementary.pdf]

Supporting Information

Transition Metal-Mediated Preparation of Nitrogen-Doped Porous Carbon for Advanced Zinc-Ion Hybrid Capacitors

Mingcheng Li[1, 2], Zheng Liu[1, *], Dan Wu[1], Huihao Wu[1], Kuikui Xiao[2, *]

[1] Key Laboratory of Low Carbon and Environmental Functional Materials of College of Hunan province, College of Materials and Chemical Engineering, Hunan City University, Yiyang, 413000, P. R. China.; limingcheng23@163.com(M.L.); wudanwd@hnu.edu.cn (D.W.); huihaowu1@gmail.com(H.W.)

[2] Key Laboratory of Carbon Materials of Zhejiang Province, College of Chemistry and Materials Engineering, Wenzhou University, Wenzhou 325035, P. R. China.

* Correspondence: liuzheng@hncu.edu.cn(Z.L.); xiaokuikui@wzu.edu.cn (K.X.)

Methods

According to the cyclic voltammetry curves, the specific capacitance of the material can be calculated using equation S1:

$$C_g = \frac{S}{2mv\Delta U} \qquad \text{(S1)}$$

where S is the area under the curve, m is the mass of the active material, v is the scanning rate, and ΔU is the voltage window. Based on the constant current charge-discharge curves, the specific capacity of the material can be calculated using equation S2:

$$C_m = \frac{I \cdot \Delta t}{3.6m} \qquad \text{(S2)}$$

Where C_m is the specific capacity (mAh g^{-1}), I is the discharge current (A), Δt is the discharge time and m is the amount of active material loaded on the stainless steel foil.

The energy density (E, Wh kg^{-1}) and power density (P, W kg^{-1}) of the zinc-ion hybrid capacitor can be calculated using equation S3 and S4:

$$E=\frac{C_g \Delta U^2}{2\times 3.6} \quad \text{(S3)}$$

$$P=\frac{3600E}{\Delta t} \quad \text{(S4)}$$

According to the CV profiles, the relationship between the cathodic and anodic peak current densities response (i) and the scan rate (v) was analyzed by employing the following equation:

$$i = av^b \quad \text{(S5)}$$

$$\log(i) = b\times\log(v)+\log(a) \quad \text{(S6)}$$

where i and v represent current (A) and scan rate (V s^{-1}), respectively. a and b are the adjustable constants[1].

The ratios of the capacitive contributions can be calculated based on the total current response (i) and scan rate (v), as shown in the following equation:

$$i(V)=i_{capacitive}+i_{diffusion}=k_1v+k_2v^{1/2} \quad \text{(S7)}$$

In equation S7, the adjustable parameters k_1 and k_2 can be determined by plotting $i(V)/v^{1/2}$ versus $v^{1/2}$ and the contributions of the surface capacitive and diffusion-dominated processes can subsequently be calculated by k_1v and $k_2v^{1/2}$, respectively.[2]

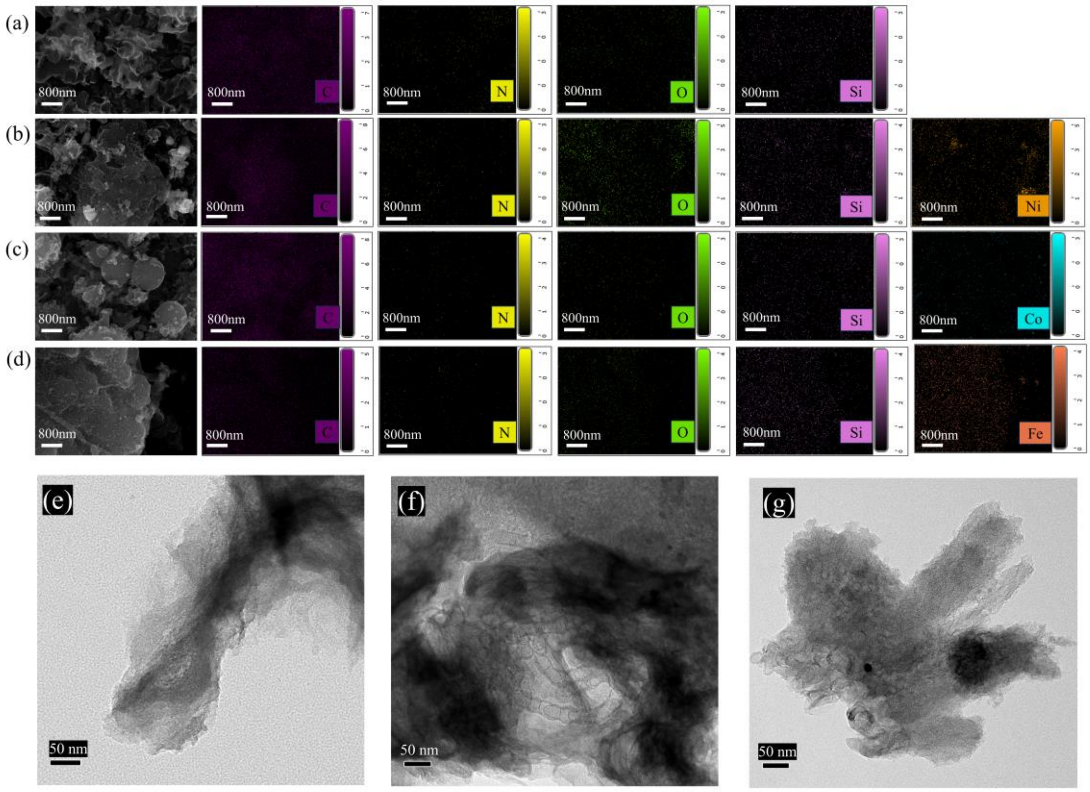

Figure S1. EDS-mapping images of (a) NDPC, (b) Ni-NDPC, (c) Co-NDPC and (d) Fe-NDPC;

TEM image of (e) NDPC, (f) Ni-NDPC, (g) Co-NDPC

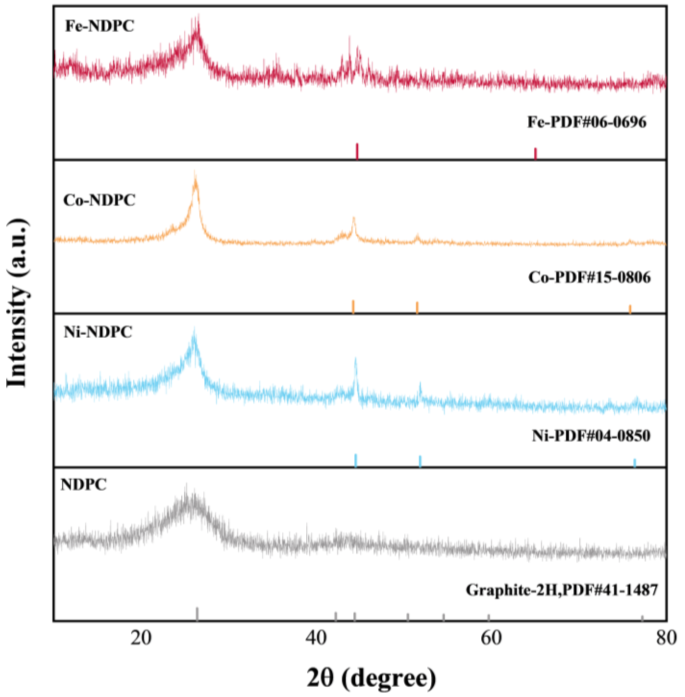

Figure S2. XRD spectra of the four sample.

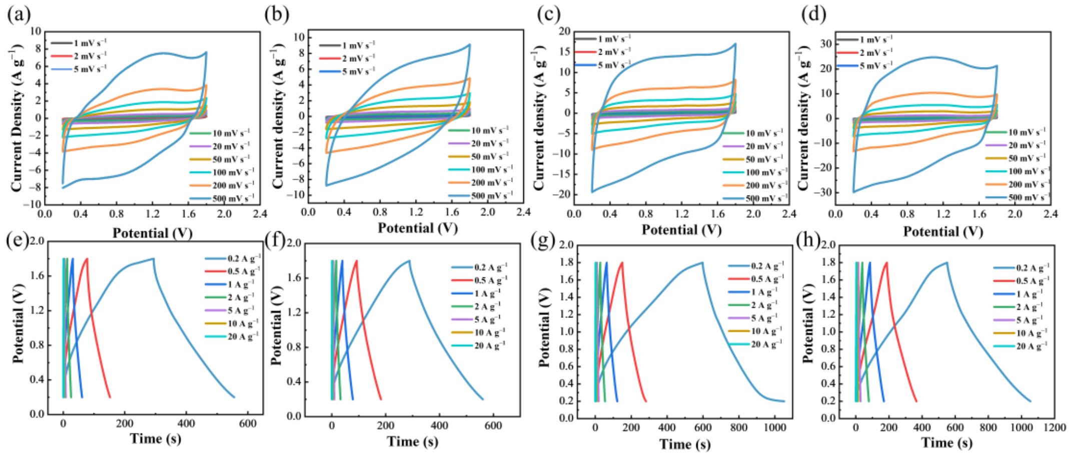

Figure S3. Cyclic voltammograms of (a) NDPC, (b) Ni-NDPC, (c) Co-NDPC and (d) Fe-NDPC at 1-500 mV s^{-1}. CP curves at different current densities from 0.2 to 20 A g^{-1} for (e) NDPC, (f) Ni-NDPC, (g) Co-NDPC and (h) Fe-NDPC

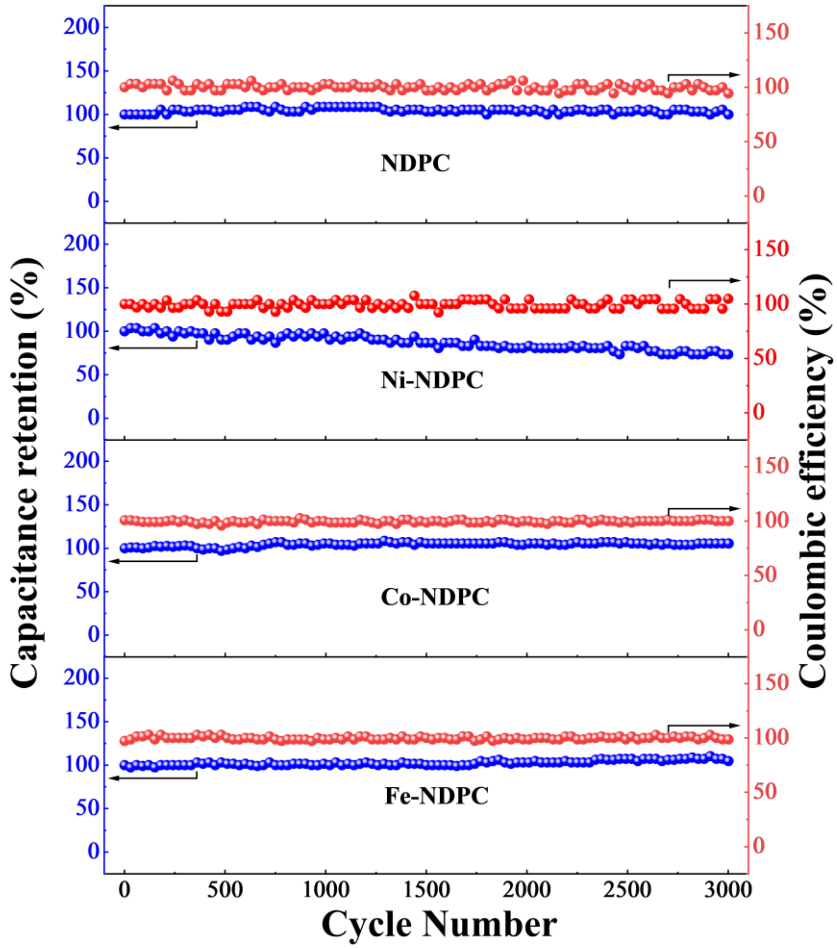

Figure S4. Cycle stability at 5 A g^{-1} of the samples.

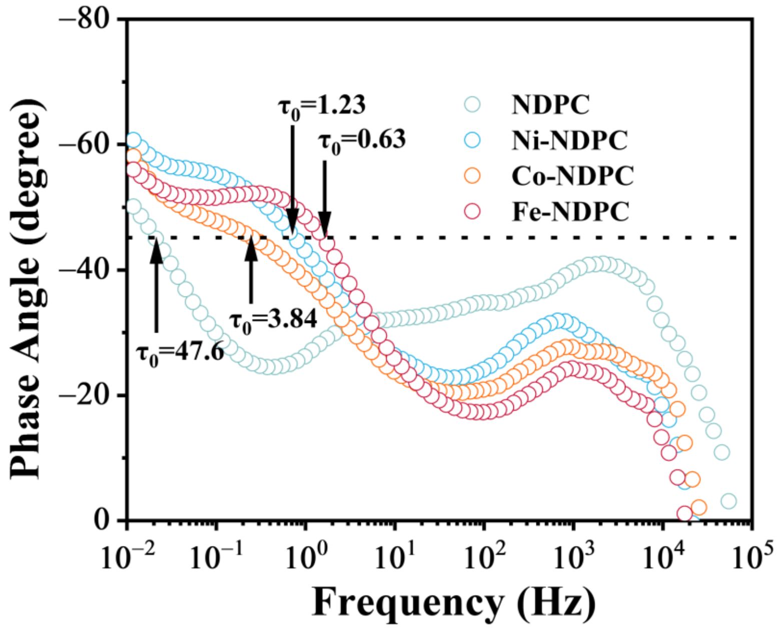

Figure S5 Bode plots of phase angle versus frequency.

Table S1. The content of various elements in the samples.

	C content (%)	N content (%)	Metal content (%)	O content (%)
NDPC	80.03	8.67	0	11.31
Ni-NDPC	64.65	3.11	7.37 (Ni)	24.86
Co-NDPC	92.9	2.41	0.33 (Co)	4.36
Fe-NDPC	76.72	4.4	2.21 (Fe)	16.67

Table S2. The proportion of carbon bonds in the samples

	C-C/C=C (%)	C-N/C-O (%)	C=O (%)	π-π* satellite (%)
NDPC	68.79	16.81	8.88	5.53
Ni-NDPC	66.92	16.17	11.01	4.47
Co-NDPC	70.76	12.31	9.95	6.99
Fe-NDPC	66.91	13.2	13.1	6.8

Table S3. The area normalized capacitance of the samples

	Area Normalized Capacitance at 1 A g^{-1} (μF cm^{-2})	Area Normalized Capacitance at 20 A g^{-1} (μF cm^{-2})	Area Normalized Capacitance at 1 mV s^{-1} (μF cm^{-2})	Area Normalized Capacitance at 500 mV s^{-1} (μF cm^{-2})
NDPC	38.64	8.14	66.55	17.43
Ni-NDPC	20.41	2.28	28.90	6.81
Co-NDPC	22.18	5.53	28.51	12.25
Fe-NDPC	22.17	7.18	30.04	13.80

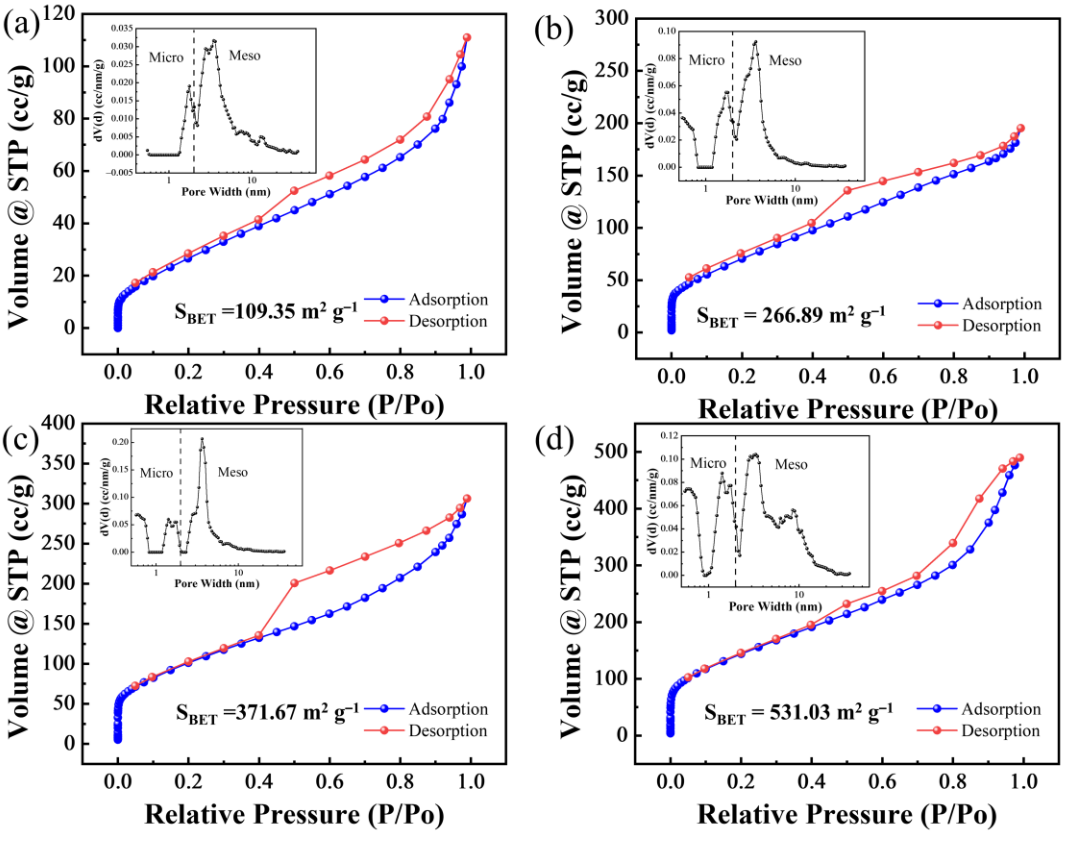

Figure S6. N_2 adsorption-desorption isotherm of (a) NDPC, (b) Ni-NDPC, (c) Co-NDPC and (d) Fe-NDPC. (The inset are the respective aperture distributions.)

References

1. Gao, P.; Shen, B.; Zhao, P.; Shi, G.; Zhao, X. Tuning the Mn^{2+}/Mn^{3+} ratio of $ZnMn_2O_4$ from spent zinc-carbon battery powder to enhance the electrochemical performance. *J. Power Sources* **2023**, *577*, 233231.

2. Gao, T.; Luo, W.; Yang, Y.; Zhou, Y.; Xu, J.; Li, N.; Li, J.; Liu, Z. Engineering hierarchically porous carbon nanorods electrode materials for high performance zinc ion hybrid supercapacitors.

Colloids Surf. A **2024**, *684*, 133057.
